# TAV-in-TAV to Rescue Suboptimal Index TAVR: The Critical Role of Anatomy

**DOI:** 10.1016/j.jaccas.2025.105343

**Published:** 2025-10-22

**Authors:** Ozan M. Demir, Christopher Cook, Samer Fawaz, Rajesh Aggarwal, Rohan Jagathesan

**Affiliations:** a Essex Cardiothoracic Centre, Mid and South Essex NHS Foundation Trust, Basildon, United Kingdom; b Anglia Ruskin School of Medicine & MTRC, Anglia Ruskin University, Chelmsford, United Kingdom

**Keywords:** paravalvular leak, TAV-in-TAV, transcatheter aortic valve replacement (TAVR), valve-in-valve

## Abstract

**Background:**

Suboptimal transcatheter aortic valve replacement (TAVR) positioning can lead to inadequate sealing between the prosthesis and the native annulus, resulting in significant paravalvular leak (PVL).

**Case Summary:**

A 75-year-old woman underwent TAVR with an ACURATE neo2 valve that was complicated by severe intraprocedural aortic regurgitation and hemodynamic instability, resulting in prompt deployment of the valve, with residual moderate PVL. Initial symptomatic relief was short-lived owing to progressive heart failure. Anatomical evaluation revealed substantial constraints, guiding the successful implantation of a high-positioned Sapien 3 Ultra transcatheter valve-in-valve (TAV-in-TAV). Meticulous coronary protection and iterative balloon postdilatation resolved PVL without coronary compromise.

**Discussion:**

This case emphasizes the critical role of detailed anatomical evaluation and precise device positioning in TAV-in-TAV procedures, particularly when managing risks associated with specific valve prosthesis features, such as ACURATE neo2 upper crowns.

**Take-Home Message:**

Detailed anatomical assessment and meticulous device positioning are fundamental for successful outcomes in complex TAV-in-TAV interventions.

## Graphical Abstract

**Figure:**
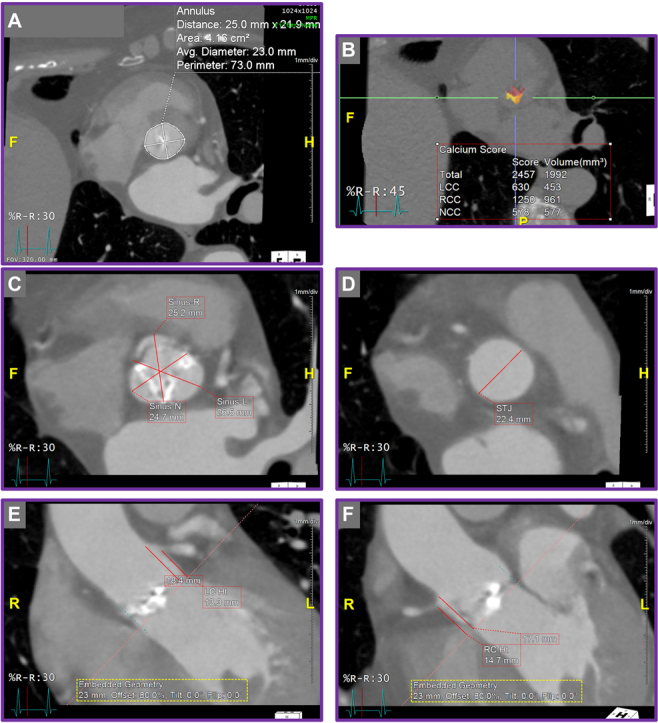

## History of Presentation

A 75-year-old woman with a medical history of hypertension, type 2 diabetes mellitus, antiphospholipid syndrome, and prior cerebrovascular accidents was referred to the regional aortic valve team for evaluation of symptomatic severe aortic stenosis. She presented with NYHA functional class III dyspnea, and transthoracic echocardiography confirmed severe aortic stenosis, with a maximum aortic velocity of 4.5 m/s, peak transvalvular gradient of 83 mm Hg, mean gradient of 47 mm Hg, and preserved left ventricular systolic function with an ejection fraction of 60%. In view of her comorbidities, the heart team consensus was that she be treated with transcatheter aortic valve replacement (TAVR).

**Take-Home Messages**

• TAV-in-TAV is an effective bailout strategy for managing PVL following suboptimal ACURATE neo2 deployment, but requires meticulous anatomical assessment and procedural planning.
• Understanding the interaction between native anatomy and both index and second valve designs is essential to address the mechanism of prosthesis failure and optimize outcomes.

**Figure 1:**
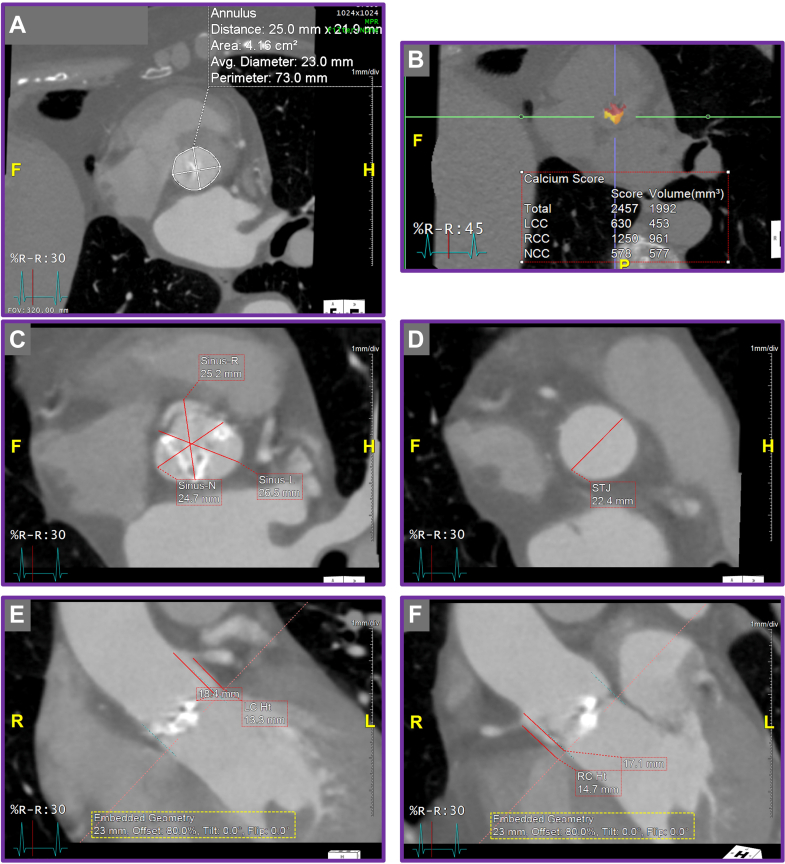
Initial Transcatheter Aortic Valve Replacement Protocol Computed Tomography (A) Annular measurements. (B) Predominant calcification on aortic valve leaflets. (C to F) Stovepipe annular configuration.

**Figure 2:**
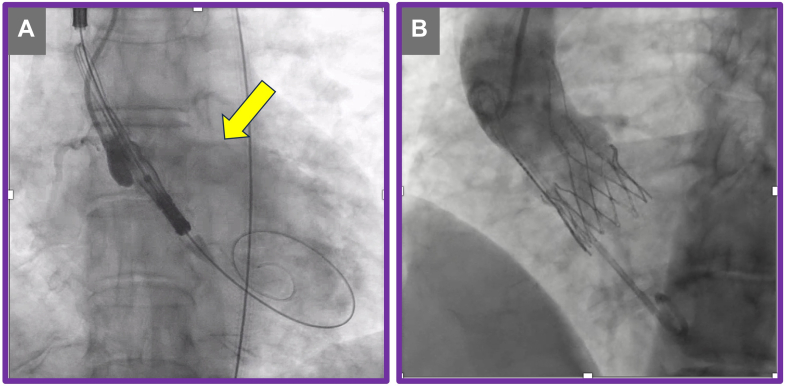
Index Transcatheter Aortic Valve Replacement Procedure (A) Severe aortic regurgitation (yellow arrow). (B) Deployment of ACURATE neo2 valve.

Initial TAVR protocol computed tomography (CT) scan demonstrated anatomical suitability for transcatheter intervention, with a stovepipe annular configuration (Figures 1A to 1F). The multidisciplinary heart team recommended TAVR using a medium-sized ACURATE neo2 valve (Boston Scientific). The procedure was performed via transfemoral access, with predilatation using a 22-mm TRUE balloon (Becton Dickinson). During positioning of the ACURATE neo2 valve at the annular level, the patient developed severe aortic regurgitation across the native aortic valve, resulting in hemodynamic instability, with a systolic/diastolic blood pressure of approximately 50/30 mm Hg, refractory to pharmacologic circulatory support. As a result, the TAVR was promptly deployed, leading to immediate hemodynamic stabilization (Figures 2A and 2B, Videos 1 and 2).

**Figure 3:**
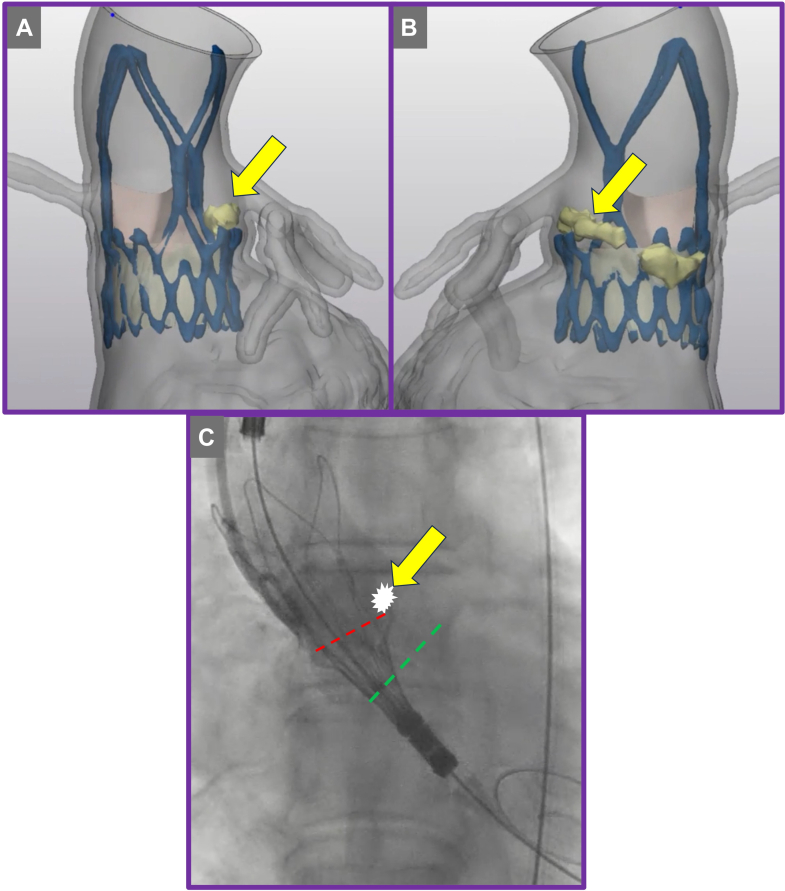
Redo Transcatheter Aortic Valve Replacement Computed Tomography Analysis (A and B) Calcification above the upper crown of the index transcatheter aortic valve replacement prosthesis (yellow arrow). (C) Annular plane (green dashed line) and misaligned deployment plane (red dashed line).

Postdeployment assessment revealed mild to moderate paravalvular leak (PVL). The mechanism of PVL was attributed to heavy calcification at the tip of the native aortic leaflet (Figures 1A and 1B) extending above the upper crowns of the implanted ACURATE neo2 valve on the left coronary cusp leaflet (Figures 3A to 3C). The combination of heavy calcification at the native aortic valve leaflet tips and emergent deployment of the transcatheter valve—together with the distinctive upper crown design of the ACURATE neo2 valve—resulted in suboptimal valve positioning and PVL.

**Figure 4:**
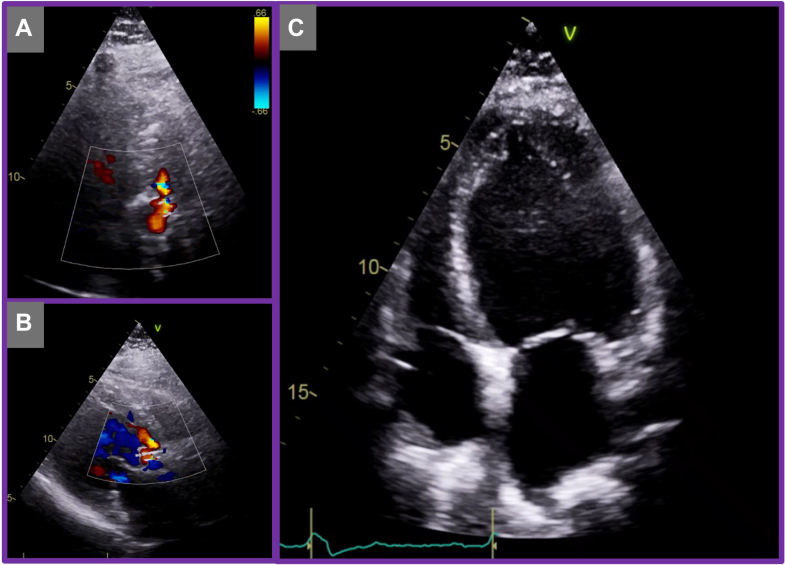
Transthoracic Echocardiography at 10 Months Post-Transcatheter Aortic Valve Replacement (A and B) Demonstrating severe aortic regurgitation. (C) Demonstrating dilated severe left ventricular systolic dysfunction.

Despite initial symptomatic improvement, the patient was readmitted with decompensated heart failure 10 months after the index TAVR. Transthoracic echocardiography demonstrated severe paravalvular leak around the transcatheter valve prosthesis (Figures 4A to 4C, Video 3), accompanied by significant left ventricular dilatation and severe systolic dysfunction. Given the progressive decline in ventricular function, the multidisciplinary heart team recommended further evaluation for a transcatheter valve-in-valve (TAV-in-TAV) intervention. A repeat CT TAVR protocol scan was subsequently performed for procedural planning.

## Past Medical History

In addition to hypertension, type 2 diabetes mellitus, antiphospholipid syndrome, and prior cerebrovascular accidents, the patient had a history of peripheral neuropathy and was a current smoker.

## Differential Diagnosis

The differential diagnosis included progressive left ventricular dysfunction due to significant paravalvular leak following suboptimal TAVR positioning as well as structural valve degeneration of the previously implanted transcatheter heart valve.

## Investigations

**Figure 5:**
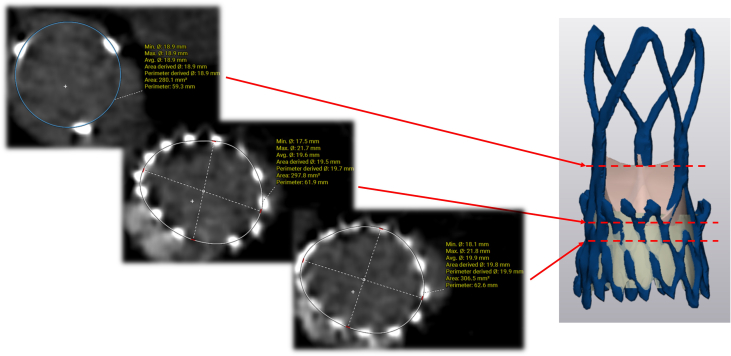
Computed Tomography Analysis Across Index Transcatheter Aortic Valve Replacement Prosthesis

**Figure 6:**
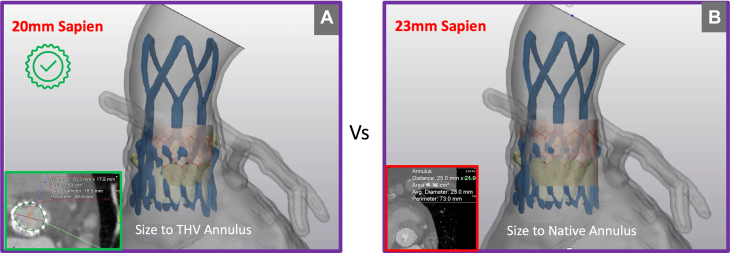
Redo Transcatheter Aortic Valve Replacement Valve Sizing Comparison of virtual valve fit for (A) 20-mm vs (B) 23-mm Edwards SAPIEN 3 Ultra prostheses.

Comprehensive postindex TAVR assessment, incorporating multiphase CT and transthoracic echocardiography, demonstrated no evidence of central regurgitation, significant underexpansion, or coaptation defect. These findings support supra-annular malpositioning—specifically interference from calcified native leaflet tips—as the principal mechanism of valve failure (Figures 3 and 5). Commissural alignment of the ACURATE neo2 was preserved, and TAV-in-TAV simulation confirmed anatomical feasibility for both 20-mm and 23-mm Edwards SAPIEN 3 Ultra (S3U) prostheses as feasible reintervention options. Given the risk of annular injury owing to the upper crown of the index TAVR valve abutting the heavily calcified tip of the native leaflet, detailed imaging-based risk assessment favored selecting the smaller, 20-mm valve to minimize annular trauma and coronary occlusion (Figures 6A and 6B). A high-position deployment strategy was planned, aligning the outflow of the S3U precisely at the base of the commissure posts, with bilateral coronary artery protection—this would enable sealing of the PVL at the level of the upper crowns of the index TAVR.

## Management (Medical/Interventions)

**Figure 7:**
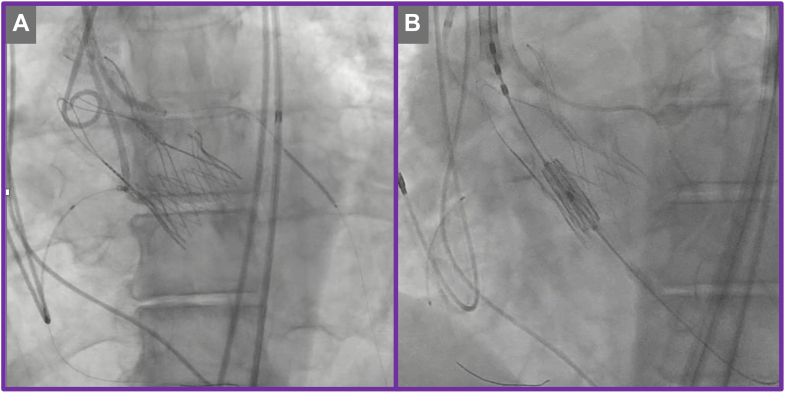
Transcatheter Valve-in-Valve Procedure (A) Bilateral coronary protection. (B) Positioning of the redo transcatheter aortic valve replacement prosthesis.

**Figure 8:**
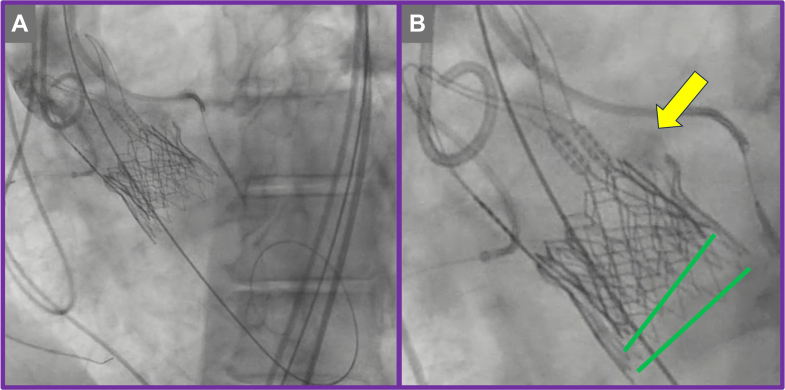
Deployment of Redo Transcatheter Aortic Valve Replacement Prosthesis (A) Initial deployment at nominal expansion. (B) Final position showing sealing skirt engagement with calcium (yellow arrow) above the index transcatheter aortic valve replacement prosthesis.

**Figure 9:**
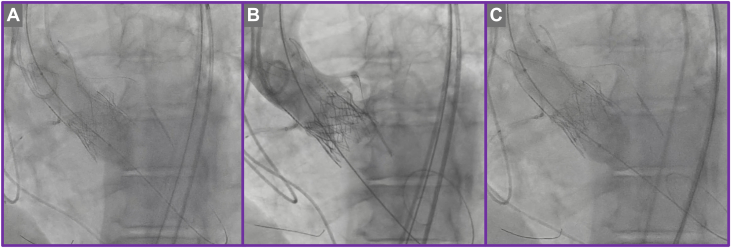
Iterative Postdilatation of Redo Transcatheter Aortic Valve Replacement Prosthesis (A) Balloon aortic valvuloplasty with 21-mm balloon. (B) Aortography showing residual paravalvular leak. (C) Further postdilatation with 22-mm balloon aortic valvuloplasty.

**Figure 10:**
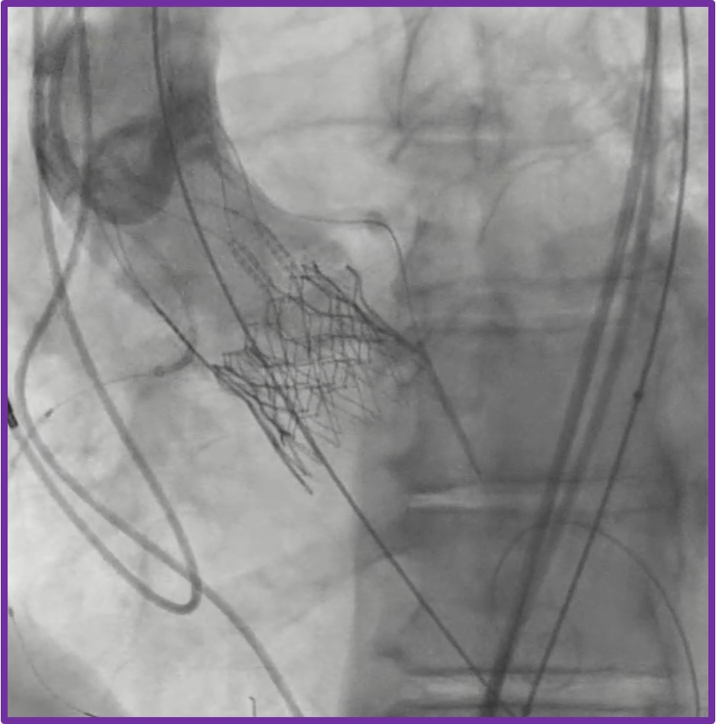
Final Result Following Redo Transcatheter Aortic Valve Replacement Demonstrating effective paravalvular leak resolution and preserved valve alignment.

The TAV-in-TAV procedure was performed via right transfemoral access. Initial aortography confirmed severe PVL localized to the left coronary cusp (Video 4). Meticulous bilateral coronary artery protection was undertaken by positioning coronary guidewires and undeployed coronary stents within the proximal-to-mid segments of the right coronary artery and left anterior descending artery, appropriately sized according to the ostial vessel caliber (Figures 7A and 7B, Video 4). This protective strategy was employed proactively to mitigate the risk of coronary obstruction during valve deployment. The index transcatheter valve was crossed using a pigtail catheter, ensuring careful avoidance of entanglement with the ACURATE neo2 stabilization arches (Figure 7A). A 20-mm Edwards SAPIEN 3 Ultra valve was then implanted in the predefined high position (Figures 7B, 8A and 8B, Videos 5 and 6). Despite precise valve deployment, significant residual PVL persisted (Video 7), owing to calcium above the upper crowns of the index TAVR, necessitating an iterative balloon dilatation strategy. Sequential postdilatations were carefully performed using incremental balloon sizes of 21-mm and subsequently 22-mm TRUE balloons (Figures 9A to 9C, Video 8), closely monitoring valve expansion fluoroscopically. This stepwise balloon dilatation approach ultimately resulted in effective sealing of the PVL (Figure 10, Video 9). Final imaging confirmed preserved coronary patency, with unobstructed flow in both coronary arteries clearly demonstrated on angiography; therefore, deployment of the prepositioned chimney stents was not required.

## Outcome and Follow-Up

Following a successful TAV-in-TAV procedure, echocardiogram demonstrated no PVL and mean aortic valve gradient of 7 mm Hg. The patient was discharged on postprocedure day 1 without complications. At 6-week outpatient follow-up, she reported marked symptomatic improvement, with no further episodes of heart failure or hospital readmission. Transthoracic echocardiography demonstrated stable prosthetic valve function and no PVL, and there was no significant change in left ventricular function at this time point.

## Discussion

TAV-in-TAV implantation is increasingly recognized as a viable treatment strategy for failed transcatheter heart valves.^1^, ^2^, ^3^, ^4^ However, performing TAV-in-TAV in the context of prior ACURATE neo2 implantation presents unique anatomical and procedural challenges that require careful preprocedural planning. The ACURATE neo2 valve is a supra-annular, self-expanding device with stabilizing arches and an upper crown that can project beyond the annular plane. Whereas this design contributes to ease of deployment and hemodynamic performance, it may complicate future interventions. Moreover, the recent ACURATE neo2 investigational device exemption study demonstrated significantly higher rates of prosthetic valve aortic regurgitation (central plus paravalvular) at 1 year compared with other commercially available TAVR protheses (mild aortic regurgitation 42.5% vs 24.8%, *P* < 0.0001; moderate 4.4% vs 1.8%, *P* = 0.0070; severe 0.5% vs 0%; *P* = 0.12).^5^ In addition, valve underexpansion and asymmetrical frame geometry have been reported in heavily calcified anatomies, contributing to suboptimal sealing and PVL—a scenario seen in this case.^6^ Given that the ACURATE neo2 is one of the main TAVR platforms used in Europe, understanding how to safely and effectively perform TAV-in-TAV for failing prostheses is crucial.

CT-based simulation and anatomical analysis, as used in this case, are essential in anticipating and mitigating procedural risks and achieving good outcomes. The decision to use a smaller 20-mm SAPIEN 3 Ultra valve in a high implant position was informed by the risk of annular injury from interaction between the ACURATE neo2 upper crown and heavily calcified native leaflet tips—a mechanism of PVL also described in recent analyses. High positioning of the SAPIEN 3, with alignment at the base of the commissure posts, aimed to optimize sealing and minimize further structural disruption—specifically positioning the sealing skirt of the SAPIEN 3 against the calcium in the left coronary cusp position (Figure 8B). Iterative postdilatation using 21-mm and 22-mm noncompliant balloons aligns with procedural best practices. Controlled expansion ensures improved valve frame conformity and leaflet coaptation without increasing the risk of annular rupture, particularly in the constrained annular settings often seen with underexpanded ACURATE neo2 frames. Coronary protection with prepositioned guidewires and undeployed stents has emerged as a prudent strategy in high-risk TAV-in-TAV cases.^7^ A multicenter observational study demonstrated that proactive coronary protection during valve-in-valve procedures significantly reduced the incidence of coronary obstruction and the need for bailout chimney stenting.^8^ In this case, although chimney stenting was not ultimately required, the prepositioned system provided critical procedural safety.

This case underscores 3 key considerations in managing failed ACURATE neo2 valves: 1) preprocedural imaging is critical, especially to assess neoskirt height, commissural alignment, and coronary take-off in relation to the valve frame; 2) device selection must be anatomy-specific, with particular attention to frame interaction and residual leaflet bulk; 3) routine coronary protection should be strongly considered in all ACURATE neo2 TAV-in-TAV cases owing to the structural risk profile of the device. Although the experience with TAV-in-TAV after ACURATE neo2 is still emerging, this case illustrates how procedural planning and anatomy-guided strategy can result in successful outcomes—even in complex settings.

## Conclusions

TAV-in-TAV can serve as an effective treatment strategy for managing paravalvular leak in select cases. However, suboptimal positioning of the index transcatheter heart valve presents unique anatomical and procedural challenges. Successful reintervention requires comprehensive preprocedural planning, including detailed assessment of anatomical constraints and device-specific features. A thorough understanding of these factors enables an iterative, anatomy-guided approach that mitigates the risk of catastrophic complications such as coronary obstruction or annular rupture.

**Visual Summary:** Case Timeline

Timeline	Events
Day 1	A 75-year-old woman with symptomatic severe aortic stenosis underwent index transcatheter aortic valve replacement using self-expanding medium-sized ACURATE Neo 2.
Day 2	Post-index transcatheter aortic valve replacement echocardiogram confirmed mild to moderate paravalvular leak. Patient had normal left ventricular systolic function.
30-day follow-up	Patient was asymptomatic and reported improved quality of life.
10 mo	Patient was readmitted with decompensated heart failure 10 months after index transcatheter aortic valve replacement. Transthoracic echocardiography demonstrated severe paravalvular leak around transcatheter valve prosthesis, accompanied by significant left ventricular dilatation and severe systolic dysfunction.
10 mo 1 wk	Second computed tomography scan of transcatheter aortic valve replacement and transcatheter valve-in-valve simulation was performed.
10 mo 2 wk	Redo transcatheter aortic valve replacement with bilateral coronary protection was performed using Edwards Sapien 3 20 mm with iterative postdilatation strategy to minimize risk of complications, in particular annular injury, during sealing of the paravalvular leak.
12 mo (postredo transcatheter aortic valve replacement follow-up)	Patient showed marked symptomatic improvement, with no further episodes of heart failure or hospital readmission.

## Funding Support and Author Disclosures

Dr Cook is a consultant for Edwards Lifesciences, Boston Scientific, and Phillips. All other authors have reported that they have no relationships relevant to the contents of this paper to disclose.

## Appendix

Video 1 Index Transcatheter Aortic Valve Replacement Positioning Complicated by Severe Aortic Regurgitation

Video 2 Aortogram Post-Index Transcatheter Aortic Valve Replacement Showing Mild to Moderate Paravalvular Leak

Video 3 Transthoracic Echocardiogram Demonstrating Severe Left Ventricular Dilatation and Dysfunction Post-Index Transcatheter Aortic Valve Replacement

Video 4 Coronary Protection Strategy During Redo Transcatheter Aortic Valve Replacement

Video 5 Positioning of Redo Transcatheter Aortic Valve Replacement Prosthesis at High Implantation Level to Address Supra-Annular Calcium

Video 6 Deployment of Redo Transcatheter Aortic Valve Replacement Prosthesis

Video 7 Aortogram Showing Persistent Paravalvular Leak Following Initial Redo Transcatheter Aortic Valve Replacement Deployment

Video 8 Postdilatation of Redo Transcatheter Aortic Valve Replacement Prosthesis Using a 22-mm Balloon

Video 9 Final Result Following Redo Transcatheter Aortic Valve Replacement With Complete Paravalvular Leak Resolution
